# “Divergent Needs and the Empathy Gap”: Exploring the Experience of Workplace Violence Against Nurses Employed in the Emergency Department

**DOI:** 10.3390/healthcare13101118

**Published:** 2025-05-11

**Authors:** Christina Koutsofta, Maria Dimitriadou, Maria Karanikola

**Affiliations:** Department of Nursing, School of Health Sciences, Cyprus University of Technology, Limassol 3036, Cyprus; cg.koutsofta@edu.cut.ac.cy (C.K.); maria.dimitriadou@cut.ac.cy (M.D.)

**Keywords:** violence, nurses, ED, emergency department, implications, workplace violence, lived experience, patients, qualitative data, content analysis

## Abstract

Background/Objectives: Violence in healthcare settings, especially in emergency departments (ED), remains an important public health issue worldwide. Thus, additional insight into the effect of these incidents into nurses’ professional attitudes, their work life and related implications to patient safety issues may be valuable. We investigated ED nurses’ living experience of exposure to workplace violence by healthcare service users, with focus on the impact on them. Methods: Following a qualitative study design, data were collected (January–June 2024) through semi-structured interviews with open-ended questions and were analyzed according to an inductive, content analysis approach. Participants provided informed consent, and data collection continued until theoretical saturation was reached. Results: The sample included six nurses. Various forms of workplace violence and its psychological, social, and professional consequences were identified. Violence was more frequently perpetrated by patients’ relatives, with verbal aggression being the most common form. A fundamental divergence in needs and expectation between patients and their family members/caregivers, on one side, and participants, on the other, revealed a pronounced empathy gap. Each group remained focused on its own priorities while struggling to recognize or accommodate others’ perspectives. This lack of mutual understanding contributed to tension that, in some cases, escalated even into physically violent incidents against the participants. A similar gap was identified between the participants’ needs and administrators’ attitudes and related policies. The failure of administrative measures to bridge this gap was described as a crucial factor in further escalating conflicts and tension in the ED. Conclusions: Further research on quality improvement projects, including all stakeholders, aiming to enhance empathy in all parties involved is proposed.

## 1. Introduction

Violence against healthcare workers is a global concern, with detrimental consequences for individuals, healthcare systems and, most importantly, for the quality and safety of provided care [1]. Allen et al. [2] defined violence as any behavior that is intentionally used to control, punish, or oppress an individual or group of individuals through denigration and fear. According to Aljohani et al. [3], seven out of ten nurses worldwide have experienced workplace violence. Their meta-analysis of 26 studies and 9072 cases of workplace violence in emergency departments (EDs) found that 72% of incidents involved verbal violence, while 18% were reported as physical violence. Family members were the primary perpetrators (52%), followed by patients (27%) and other relatives or friends (21%). The prevalence of verbal violence was 77%, indicating the widespread nature of the phenomenon [3].

Workplace violence manifests in different forms. Physical violence refers to any act of physical aggression that causes harm or injury through direct contact, bodily secretions, or thrown objects, including biting, scratching, punching, kicking, stabbing, or threatening gestures [4]. Verbal violence involves spoken or written language used aggressively to intimidate, threaten or humiliate a target person or group. This includes profanity, shouting, insults, derogatory comments, inappropriate commands and sarcasm [4]. Psychological violence, also known as emotional abuse, includes any non-physical abusive behavior aimed at manipulating or coercing an individual into specific actions [4]. Unlike physical and verbal aggression, psychological violence is often covert, making it harder to detect. It manifests in behaviors such as silence and ignorance, threats, guilt induction and emotional blackmail, and critical attitudes [5]. Psychological violence may also occur indirectly, such as through witnessing violent behaviors directed at others, or through exerting pressure to others to conform to coercive norms [6].

The World Health Organization in the 2000s reported that 8% to 38% of nurses globally had experienced physical violence in their workplace [5], and this prevalence is still increasing [3]. Notably, almost six out of ten workplace violence survivors have considered leaving their jobs [7]. This issue has significant effects, not only for individual nurses by leading to emotional distress and job dissatisfaction, but also for healthcare systems, since it is linked to diminished quality of care and reduced patient safety [8]. Specifically, workplace violence in ED has been associated with increased absenteeism, high turnover rates, and decreased staff morale, all of which directly affect patient outcomes [9].

Nevertheless, exposure to violent behaviors in the workplace directly affects nurse survivors in personal, social, and professional level including enduring and traumatizing consequences [7]. Although prior research underscored the immediate consequences of workplace violence, its broader impact across multiple levels of nurses’ lives remains unexplored [8]. To effectively address workplace violence, a comprehensive understanding of its manifestations, consequences and coping mechanisms is essential. This is of paramount importance not only for ensuring nurses’ well-being but also for maintaining high standards of quality and safety of patients’ care and healthcare organizations’ long-term sustainability [6].

Previous research highlighted multiple contributing factors to workplace violence against nurses [7]. A study in the Republic of Cyprus (RC) revealed as the most significant organizational risk factor long patient waiting times [10]. Additionally, individual risk factors, such as alcohol intoxication, substance use and mental illness were frequently cited as triggers for violent behaviors in EDs [10]. One of the most pressing concerns in addressing workplace violence was severe underreporting. According to Vezyridis et al. [10], almost seven out of ten workplace violence incidents remained underreported, while 74% of participating nurses in the study believed that violence was an inherent part of their work. This normalization of violence contributed to ongoing exposure and further psychological harm. Nevertheless, exposure to workplace violence has severe implications for nurses’ mental, physical, professional and social well-being [10]. A cross-sectional study in Greece on workplace bullying reported that a significant proportion of participating nurses had experienced verbal abuse, with 77.6% of these incidents involving a patient’s relative [11]. This study further assessed the cognitive, emotional and behavioral consequences of workplace violence on the participants, highlighting the severity of its impact on their well-being [11]. Yet, additional insight into the effect of these incidents into nurses’ professional attitudes and their work life and related implications to patient safety issues may be valuable.

Indeed, despite the severity of workplace violence in EDs, qualitative research on the topic remains scarce, particularly in the Mediterranean and South European region in the post-COVID era. The present study qualitatively aimed to explore the experience of workplace violence in nurses employed in the EDs in the RC in the post-COVID era, focusing (a) on their perceptions and interpretations of the phenomenon, (b) the impact of workplace violence on them, and (c) the coping strategies used to mitigate its impact. Existing quantitative studies have primarily focused on specific dimensions of the phenomenon, such as prevalence rates and risk factors [10,11]. However, to our knowledge, no qualitative studies have explored the experiences of ED nurses in the Eastern Mediterranean or South European regions in the post-COVID era.

Recent international research has addressed the topic from different angles. The study by Whalen et al. [12] in the USA qualitatively assessed barriers and facilitators regarding reporting of workplace violence in EDs. While their findings provided valuable insight into effective reporting, they did not explore the broader impact of workplace violence on nurses’ experiences. Thomas et al. [1] explored the organizational aspect of workplace violence in Australia, but their qualitative data from 18 ED nurses were collected in the pre-pandemic period. Similarly, Hsu et al. [13], in their qualitative study of 10 Taiwanese ED nurses, explored the effects of workplace violence against nurses on quality of care and post-incident support. However, this study was also conducted before the pandemic.

Given the documented significant increase in verbal, psychological and physical assaults against frontline healthcare personnel, including ED nurses, since the onset of the COVID-19 pandemic [14], there is a need for further qualitative exploration of the topic in the post-pandemic era. A qualitative investigation into workplace violence in EDs in the Republic of Cyprus would provide valuable insights for both local and the broader European region. Specifically, a deeper insight into the dynamic of the phenomenon is expected to inform policy transformations, institutional strategies, and broader preventative measures against workplace violence, thus enhancing ED nurses’ quality and safety of work life. The latter is deemed as a crucial factor linked with nurses’ retention and engagement into the profession [7,9]. By retaining engaged nurses into the profession, better nurse–patient ratios and increased quality of care are achieved, as well as safeguarding optimal work conditions and clinicians’ well-being, all of which are linked to better clinical outcomes [8].

## 2. Materials and Methods

### 2.1. Aim

The aim of the present study was to explore the experience of workplace violence among Greek-speaking nurses working in the ED in the RC. The focus of the study was on the participants’ perceptions and interpretations of the phenomenon, with emphasis on its impact and coping mechanisms.

### 2.2. Design

A qualitative methodology, encompassing conventional content analysis approach and an inductive method was applied. Conventional content analysis is particularly appropriate for exploring under-researched topics, or a phenomenon with limited prior data [15]. Unlike methods such as thematic analysis, which often rely on pre-existing theoretical constructs, or phenomenology, which focuses on capturing the essence of lived experiences and phenomena from a philosophical standpoint, conventional content analysis allows themes to emerge directly from participants’ narratives. This makes it a more relevant to the aim of the present study, which was focused on identifying patterns and contextual dimensions in ED nurses’ experience on workplace violence in Mediterranean and South European countries in the post-COVID period [16,17].

The “CASP” [Critical Appraisal Skills Programme] qualitative checklist was applied to ensure the methodological quality of the present work, and the COREQ [COnsolidated criteria for REporting Qualitative research] checklist was used for the reporting of its protocol [please see Supplementary Materials].

### 2.3. Target Population and Sampling

The target population of the study was nurses employed in the ED of private and public hospitals in the RC. Aiming to gain access to the entire target population, the study was advertised via a variety of methods, including promotion by the Cyprus Nurses and Midwives Association, posting at social media and posters displayed at hospitals and national congresses. The extensive promotion of the study within the target population ensured its visibility and accessibility to the entire nursing population of emergency care, thereby facilitating inclusive participation and partial data saturation. Moreover, data saturation was further enhanced since the participants (a) were drawn from both public and private clinical settings, (b) possessed a wide range of clinical experiences, ranging from novice to highly experienced ED nurses, and (c) came from diverse educational backgrounds.

The purposeful sampling method was implemented according to the following inclusion criteria: (a) employment in the ED, (B) being exposed to any form of violent behavior from a patient or a patient’s companion according to participants’ reports, (c) full comprehension of the study’s objectives and procedures, as this was assessed at the first meeting with the main researcher (CK), (d) written and oral informed consent to participate in the present study, (e) adequate ability to reflect on and communicate personal experiences with the researcher, according to the depth of the narrative of the first interview. Nurses who had experienced violent behavior from colleagues were excluded. There were no time-specific inclusion/exclusion criteria regarding the time since exposure to workplace violence.

The sample size was finalized to 6 nurses based on the theoretical saturation criteria determined through the simultaneous collection and analysis of data. Specifically, saturation was defined as the point at which no new themes or subthemes emerged from the data and thematic redundancy was observed across interviews. This determination followed established qualitative research standards suggesting that studies with a focused aim and a homogeneous sample may reach saturation with a relatively small number of participants [18]. Indeed, all six participants were ED nurses within similar organizational contexts and described converging experiences of workplace violence. After the fifth and sixth interviews, analysis yielded no additional codes, and the categories already identified were consistently reinforced

Recruitment was completed when no more novel themes emerged according to the data collected analysis. At that point in time, study advertisement and recruitment strategies stopped. All those who contacted the researcher fulfilled the inclusion criteria and subsequently were included in the sample (response rate 100%). There were no dropouts from the study’s sample.

### 2.4. Data Collection

Those who willingly contacted the main author (CK) were informed of the objectives and procedures of the study and were asked to be assessed on the degree to which they met the study’s inclusion criteria. Following their informed consent, semi-structured, personal interviews were performed by CK (R.N., Bsc, Msc) for their data collection. This took place from January to June 2024. Interviews were conducted exclusively in Greek, since according to national regulations, certified fluency in Greek is required for nursing practice in the RC.

Data were collected through an interview guide encompassing open-ended questions; this was developed by the researchers after an extensive literature review [2,3,16,17], based on the aim and objectives of the study. Each participant took part in two interviews, spaced 2–3 months apart, each lasting between 1 and 1.5 hours. The 2–3-month interval between interviews was maintained to allow for preliminary analysis of the first-round data and to facilitate reflective, focused second interviews, thereby enhancing the study’s rigor and confirmability [15]. The second interview served as a form of member checking, aimed at confirming the preliminary analysis of the first interview and enhancing the study’s trustworthiness. This follow-up allowed participants to reflect on the researchers’ interpretations, clarify ambiguities, and provide additional context where needed. This approach was informed by Munhall’s [19] criteria for rigor in qualitative research, particularly in terms of establishing resonance and reasonableness in the findings.

Based on the participants’ preferences, the interviews were conducted online, or in a place chosen by the participants which was characterized by privacy. The following open-ended questions were included in the interview guide: 1. Please, describe to me your personal experience of incidents of violence in the ED originated from patients and/or their companions. Have you ever seen these incidents happen to others? 2. If you had such an experience in what form was this? 3. What are your feelings and thoughts about these incidents? 4. Have there been any changes in your life since your exposure to these incidents? If yes, can you give me an example? 5. How have you been coping with the impact of these incidents in your life? 6. Is there anything else that you would like to discuss?

A set of demographic data were also collected at the end of the first interview, and a data sheet was used for this purpose. Specifically, the participants were invited to provide information regarding their age, gender, and years of experience in the nursing profession, as well as in the ED.

### 2.5. Ethical Issues and Statements

Interviews were conducted with confidentiality and the use of pseudonyms, ensuring the anonymity of the participants during data collection and analysis. If participants voluntarily disclosed their real name during the interview, the incidence was treated with strict confidentiality. Each participant was interviewed individually. Prior to each interview, a consent form was signed by the participants, thereby consenting to the management of their data according to the scope of the study by the research team, as well as to the recording of the interview. The consent form was accompanied by an information leaflet, which was provided to ensure comprehensive understanding of the study’s objectives and procedures by the participants. The interviews were conducted during participants’ free time to ensure confidentiality, minimize potential stigma, and prevent interference with their professional environment. It should be noted that during the interview, only CK and the participants were present.

Given the sensitive nature of discussing workplace violence, special attention was paid to monitoring and managing potential psychological distress. The interviews were conducted by the main researcher, an experienced nurse with advanced training in clinical communication and psychological assessment. During each interview, participants were continuously monitored for signs of emotional distress and were reminded that they could pause or withdraw from the interview at any time without consequence. To further support participants’ well-being, they were provided with a list of accessible public mental health services. This included contact information for affiliated mental health professionals available for follow-up if needed. Although none of the participants requested such support, several described the interview experience as therapeutic. These precautions were in line with ethical best practices and contributed to the trustworthiness of the study.

The research protocol was approved by the Cyprus National Bioethics Committee (EBK EΠ 2023.01.219). All procedures of the study fully comply with the Helsinki Declaration of 1975, as revised in 2008. All data (personal, demographic, educational/ vocational) collected and presented in the study were drawn from a population of 1,000,000 individuals and the subgroup of emergency care nurses (approximately 400) making it impossible to identify any participant. Moreover, the participants regularly confirmed their willingness for participation in the next phase of the study. Additionally, they were given a list of the public mental health services and the clinicians affiliated with the research team to provide support in cases of distress due to exposure to the violent incident.

### 2.6. Data Analysis

To ensure the rigor, trustworthiness and transferability of the study results, the triangulation method for data analysis was employed. Specifically, the content analysis team comprised the principal researcher (CK) and three academic collaborators (MK, MD and MM), who are well educated and experienced in qualitative data analysis. They independently analyzed each interview before collectively reviewing and validating the codes and themes generated by each researcher. This approach enhanced transparency also facilitated the integration of diverse perspectives and prevented the loss of any data, thereby ensuring the credibility of the results [15]. Overall, these peer debriefing sessions contributed to reflexivity and analytical rigor.

The content analysis process commenced immediately after transcribing one or two interviews. Prior to commencing the content analysis, the researcher transferred the recorded interview into printed form, and checked twice the accuracy of the matching of the content between the recorded and the printed material. Through collaborative efforts, code groups were established, reflecting the participants’ common interpretations, descriptions and perceptions. For consistency in terminology, “violence” was used interchangeably with “aggressive behavior” as an umbrella term for psychological, verbal, and physical violence [5]. Aggressive behavior was defined as any intentional act aimed at causing harm to another individual [5]. A witness to violent behavior (whether verbal, psychological or physical) was defined as a person who was indirectly exposed to such behavior, while a survivor of violent behavior was defined as an individual who was directly exposed to it [8]. The following characteristics of aggressive behavior were coded: severity, intensity, mode of expression. “Experience” was categorized as (a) thoughts and perception (psychological processes of meaning-making), (b) behaviors (practical actions), and (c) emotions (affective response to experiences) [4].

These codes facilitated the emergence of key categories and themes. Descriptive definitions were then developed for each category, theme, and further emerging codes, ensuring clarity in data selection and interpretation. Initial categories and themes were formulated directly from the data, guided by the pre-established definitions. Yet, by following an inductive approach, the analysis was not restricted by a pre-existing theoretical framework. Subsequently, levels of abstraction were identified, allowing for the development of additional codes, categories, and themes, all grounded in the same data-driven framework. Continuous peer debriefing throughout the coding and theme development process was conducted, supporting reflexivity and consistency in interpretation. However, no external audit was undertaken.

Moreover, as part of the analytical process, the research team systematically reviewed the data for contradictory or disconfirming evidence. However, the participants’ experiences showed a high degree of convergence, and no substantial contradictory cases were identified. This consistency may be attributed to the shared professional context, roles, and challenges experienced by the participants.

Overall, confirmation of the study’s rigor and trustworthiness followed the criteria of Munhall [19] (Table 1).

## 3. Results

### 3.1. Participants Socio-Demographic Characteristics

The sample consisted of two female and four male participants. Their marital status varied, with three being married (N = 3) and three unmarried (N = 3). The participants were from the Republic of Cyprus (N = 5) and Greece (N = 1). Their ages ranged from 25 to 39 years. Their work experience in the ED spanned from a minimum of two years to a maximum of 12 years. All participants followed a rotating schedule, including morning, afternoon, and night shifts. Three of them held a master’s degree in nursing. Their permanent residence was in three major urban areas of the Republic of Cyprus.

### 3.2. Participants’ Narratives

By delving into participants’ experiences, the present findings revealed the various forms of workplace violence against nurses and its psychological, social, and professional consequences. Violence was more frequently by patients’ relatives rather than by the patients themselves, with verbal aggression being the most common form.

Participants’ narratives revolved around the inherent divergence in needs between patients and their family members/caregivers, on one side, and participating emergency nurses, on the other: *“[…] they [patients/relatives] believe that they (patients) need to be examined immediately, and I think this puts patients in a state where they feel they need our attention right away—whether that’s actually the case or not.” (Haris, 6 years of work experience).*

This divergence extended beyond the needs themselves to the way they were expressed, often leading to friction and misalignment in the high-stress ED environment: *“[…] They usually lash out. One of the most common things is banging on doors, but other times, they’ll start swearing at you.” (Minas, 11 years of work experience)*.

A central aspect of this phenomenon was the pronounced empathy gap, since each group remained largely attuned to its own priorities while struggling to fully recognize or accommodate the perspectives of others. This lack of mutual understanding appeared to contribute to tension that, in some cases, escalated into violent episodes against the participants: *“[…] why won’t you just let me do my job properly? […] And then you come and get in my way, threatening me—physically or verbally.” (Ioannis, 2 years of work experience).*

Furthermore, an additional layer of divergence and subsequent empathy gap was also central to participants’ narratives, this time between the participants’ needs, on one side, and administrators’ attitudes and related policies, on the other: *“[…] You face psychological harassment from supervisors and higher-ups—like, as if the violence from outsiders wasn’t enough, we also have to deal with it from within.” (Haris, 6 years of work experience).*

The failure of current administrative measures to bridge this gap was identified as a crucial factor in escalating tension within the ED and failure to support nurses. This failure not only aggravated workplace violence but also compounded participants’ distress and professional strain: *“I’ve reached the point many times where I think about switching department or even quitting the job […]” (Anna, 7 years of work experience).*

Overall, inadequate administrative interventions, lack of support, and ineffective preventive strategies further intensified the psychological and professional toll of workplace violence on the participants: *“I choose to spend my free time at home because I need more time to myself now than I did before working in this department [ER].” (Ioannis, 2 years of work experience).*

Five key categories emerged: (1) the surreal experience of workplace violence: forms, interpretations, and characteristics; (2) experiencing workplace violence: the traumatic and multifaceted impact on the participants; (3) coping with workplace violence and strategies for de-escalation; (4) administrative failure to manage workplace violence and the hidden dimensions of violence against the participants; and (5) participants’ expectations and proposed interventions.

The core theme, the categories and the themes/sub-themes of participants narratives are presented in Table 2.

#### 3.2.1. The Surreal Experience of Workplace Violence: Forms, Interpretations, and Characteristics

Participants described workplace violence against nurses in ER as a complex and multidimensional phenomenon that profoundly impacted their personal and professional well-being.

- Participants’ Awareness of Workplace Violence
  Their narratives revealed a deep awareness of the various forms of workplace violence, distinguishing between direct exposure and witnessing violent incidents: *“Personally, I have not experienced physical violence, but I have witnessed an incident of physical violence.”* (*Christiana, 2 years of work experience)*
- The Surreal Nature of Workplace Violence
  A striking theme that emerged was the “surreal” nature of workplace violence. The participants described their experience as shocking and disorienting, evoking disbelief and dissociation: *“I was caught off guard [by this behavior] […] and I thought to myself, ‘is this really happening to me right now?” […]”(Christiana, 2 years of work experience)*
- Patterns and Forms of Workplace Violence
  Participants’ accounts revealed that workplace violence often followed distinct patterns, with incidents escalating over time and occurring frequently, reinforcing its prevalence in ER settings. The most common perpetrators were patients and their accompanying relatives, with verbal violence in the form of hostile comments, threats, and degrading language, being the most frequently encountered: *There were many times I found myself arguing with people in the ER because they get furious […].”(Harris, 6 years of work experience)*
  In addition to verbal violence, psychological violence such as indirect threats and intimidation, was frequently described. These threats, particularly insinuations about filing complaints to hospital administrators, caused deep emotional distress and intense fear: *“[…] and then there’s the fear that they [healthcare service users] might call and complain to the hospital administrators “Christiana, 2 years of work experience].* Although less frequent, physical violence had even more severe consequences: *“Her behaviour and speech were quite aggressive, and when my colleague tried to escort her out, she scratched his hand, digging her nails into his skin.” (Christiana, 2 years of work experience)*
- Violation of Boundaries: A Profound Lack of Empathy and Respect
  Workplace violence was not limited to physical attacks; participants also reported frequent violations of personal, professional, and ethical boundaries. Such behaviors demonstrated a broader lack of empathy and respect not only for the nurse participants, but also for their professional role, and the patients they cared for. Indeed, most of the time, these violations directly disrupted care for other patients: *“[…] relatives repeatedly tried to interfere with our work, constantly interrupting us—even when we were attending to other patients.” (Haris, 6 years of work experience)*
- Normalization of Workplace Violence
  Furthermore, the recurrent nature of WV resulted in its normalization, making it an expected, almost routine part of nursing work: *“[…] the threats are too many and happen very often. […] I wouldn’t say on a daily basis […] but at least once a month […].” (Christiana, 2 years of work experience)*
- Persecution, Institutional Neglect and Lack of Protection
  Despite its widespread prevalence across emergency healthcare settings, participants felt isolated in their experiences, primarily due to a lack of institutional support and protection. Specifically, the participants expressed frustration over administrative inaction, leaving them feeling vulnerable and unsupported: *“[…] There was no arrest, no further actions—nothing. And we just carried on as if it were an ordinary day. […] He [the perpetrator] later came to apologize, but of course, I wasn’t satisfied with just an apology because I had been physically attacked […].” (Leonidas, 12 years of work experience)*
- Contributing Factors to Workplace Violence
  Individual-Level Risk Factors. Participants described several personal characteristics that heightened vulnerability to violence, including gender, physical stature, and lack of experience. Discrepancies between how violence was perceived by staff participants versus administrators further contributed to underreporting and insufficient intervention: *“I always noticed that women were more often targeted for physical violence*.” (Minas, 11 years of work experience)
  Patient-Related Triggers. Patient-related factors were consistently identified as primary drivers of violent behavior. This included intoxication, disregard for hospital procedures, unrealistic expectations for care and general distrust toward medical professionals. Cultural misunderstandings and low socioeconomic status exacerbated these tensions: *“People don’t take security seriously because they know the hospital won’t throw them out—they’ll still be treated.” (Anna, 5 years of work experience)*

#### 3.2.2. Experiencing Workplace Violence: The Traumatic and Multifaceted Impact on the Participants

The study findings revealed that the impact of workplace violence extended far beyond immediate physical threats, profoundly affecting participants at personal, social and professional level.

- Personal-Level Impact: Mental, Physical and Psychological Effects
  Mental Effects. Workplace violence had severe mental, physical, and psychological effects on participants. The reported mental difficulties included sleep disturbances, changes in appetite, cognitive difficulties such as problems in concentration, and reduced productivity. Participants also turned to substance use as a coping mechanism: *“[…] The excess tension leads to sleep disturbances or the urge to consume alcohol in an attempt to relax.” (Christiana, 2 years of work experience)*
  Physical Effects. Physically, chronic stress and mainly the fear of provoking violent reactions from patients and their families led participants to neglect basic biological needs. The intense pressure and the unrealistic expectations the participants faced by patients and their relatives were evident: *“[…] Hours would pass without us taking a break, without me being able to drink some water or go to the bathroom-there is no time […] and If they saw us stepping out for a break, they would get angry and start yelling, believing that we were wasting time and causing delays in our work.” [Anna, 7 years of work experience].* The physical strain of being unable to take even short breaks resulted in exhaustion, burnout and emotional distress: *“ I was constantly exhausted and hopeless […] (Anna, 7 years of work experience)*
  Psychological Effects. Beyond the immediate impact of the fear of verbal and psychological violence on compromising participants’ physical health and self-care, the narratives also highlighted how these behaviors violated participants’ basic work rights. Most importantly, patients’ and relatives’ misinterpretation of essential breaks as negligence rather than a fundamental right to self-care revealed an empathy gap. This lack of understanding fostered hostility and added unnecessary stress to an already demanding job. Ironically, depriving participants of basic needs may have jeopardized patient care, as fatigue may increase the risk of errors. Additionally, hopelessness, learned helplessness and avoidance behaviors were also evident, exacerbating participants’ psychological distress. Repeated exposure to verbal aggression and psychological coercion from patients and families not only directly harmed the participants but also reinforced a cycle of learned helplessness: *“[..] these things happen every day, and we’ve reached a point where we filter them out and just take them for granted.” (Haris, 6 years of work experience).* Over time, they stopped asserting their needs, accepted mistreatment as inevitable, and experienced deeper exhaustion, stress and burnout: *“I leave work feeling so overwhelmed, as if they have completely drained the life out of me.” (Christiana, 2 years of work experience)*
  Beyond persistent tension, intense pressure and emotional exhaustion, the participants’ narratives underscored enduring fear, anxiety and trauma: *“I was constantly living in fear […] constantly feeling fear and anger […]”* (Haris, 6 years of experience) Specifically, it was not only the threat to their physical integrity, but also the experience of extremely brutal language linked with awfully violent images was traumatic: *“[..] he said the epic line that I’ll never forget! It was so traumatic [..] that he would catch me, put me on the hood of his car, and drag me around the area, until my face melted on the road.” (Anna, 7 years of work experience)*

Certainly, the participants were subjected to verbal threats of physical violence, which were both dehumanizing and life-threatening. These threats had a deep psychological impact, demonstrating a profound sense of helplessness, fear, and devaluation of the participants’ dignity. Such traumatic experiences were expected to have long-lasting effects on their mental health. As mentioned earlier, the normalization of violence in the participants’ work environments was evident, suggesting that such experiences had become so common that they were almost expected, thus indicating how routine such aggression had become. This also hinted at the emotional exhaustion and desensitization that may occur when such violence becomes part of the everyday experience.

Moreover, these experiences led to persistent fear, heightened suspicion and difficulty in trusting others, even in the participants’ personal life: *“I’ve become suspicious of every stranger now. Even in my personal life, I see people very differently. (Haris, 6 years of work experience).*

Impact on Self-Perception, Personal Values, and Morality. Repeated exposure to workplace violence significantly undermined participants’ self-esteem, ethical values, and professional identity. The participants described a profound decline in self-confidence, leading them to question both their clinical competencies and their moral integrity. These experiences resulted in deep disillusionment, self-doubt and a gradual erosion of self-worth, leaving the participants grappling with feelings of worthlessness.: *“[…] That I am nothing—I had such a feeling many times.” (Christiana, 2 years of work experience)*.

Recurring violent incidents not only eroded professional confidence but also fractured the participants’ perception of personal identity, making them feel disconnected from their own sense of existence.: *“There were many incidents [of violence], from patients’ relatives, mostly. And […] that was one of those that made me feel completely lost, made me feel like a loser.” (Ioannis, 2 years of work experience).*

Frequent exposure to workplace violence contributed to a cycle of self-doubt and moral questioning. The participants expressed internal conflict, wondering whether they were able to meet their professional and ethical standards in such a challenging environment. Over time, these experiences seemed to blur the boundaries between professional expectations and personal values, making it harder for them to reconcile the two.

- Social-Level Impact
  The impact of workplace violence extended into participants’ social lives, straining personal relationships and fostering isolation. The participants described increased conflicts, changes in attitudes, such as heightened suspicion and difficulty maintaining familial and social connections due to emotional exhaustion, while guilt was also evident: *“[…] I would go home to my family, vent my frustrations, and that’s when the fatigue and hardship would surface. […] It was unfair for them to always hear my complaints […].” (Anna, 7 years of work experience)*
  The participants clearly underlined withdrawal from social interactions altogether, further deepening their isolation: *“I rarely go out anymore […] I need more time to myself now than I did before working in this hospital department (ER).” (Ioannis, 4 years of work experience)*
- Professional-Level Impact
  Professional Competence. Exposure to workplace violence and the resulting learned helplessness profoundly affected the participants’ self-confidence, career satisfaction, professional identity, and ability to provide effective patient care. Confidence in their professional competence and ethical standards was notably diminished: *“This experience brings down your morale and self-confidence. When someone tells you ‘you can’t do anything,’ it’s clear that in the end, you won’t be able to do anything.” (Ioannis, 4 years of work experience)*
  Participants also described questioning their own professional competencies: *“I felt terrible; I reached a point where I started doubting my own abilities. Am I not a good enough nurse? Why am I being treated like this? I started thinking that maybe it’s something I’m doing wrong.” (Anna, 7 years of work experience)*

Repeated exposure to violence triggered a crisis of professional identity, leading participants to internalize external criticism and doubt their skills and competencies, potentially resulting in burnout or disengagement from the profession. This self-doubt appeared pervasive and systemic, further eroding their confidence.

Professional Identity. Additionally, participants frequently reported the devaluation of their professional role, particularly when they were expected to assume responsibilities outside their clinical duties, such as acting as security personnel or mediators in violent situations: *“It’s not even part of our duties, trying to protect our colleagues. In this way, we end up acting as security personnel because there’s no one else to protect us.” (Leonidas, 11 years of experience)*. The ethical and professional dilemmas faced by the participants were evident, as their roles were redefined due to organizational failure or insufficient support. Being tasked with non-clinical responsibilities not only devaluated their professional identity but also conflicted with their primary responsibility to provide patient care.

Furthermore, aiming to de-escalate aggression, participants described modifying their communication style: *“Over the years, you try to avoid it diplomatically. You might need to raise your voice. Often, you have to stand your ground.” (Minas, 12 years of experience).* This shift from compassionate caregiving to defensive figure highlighted the participants’ emotional and moral strain of managing conflict. The constant navigation of this dual role contributed to emotional exhaustion and decreased job satisfaction

Ethical and Emotional Strain. Beyond professional and identity challenges, ethical concerns became apparent in the form of secondary victimization by colleagues: *“ […] and after a hard shift, instead of receiving a thumbs-up, they judge you and call you names […]” (Haris, 6 years of work experience).* A lack of support from colleagues and administrators, combined with criticism and marginalization of the violence survivor, further exacerbated the emotional burden of experiencing workplace violence. This absence of solidarity and empathy among the participants weakened team cohesion, a crucial parameter in effective patient care. The resulting sense of injustice left participants feeling isolated and unsupported.

Disruption of Healthcare Processes and Patient Care. Workplace violence also disrupted healthcare processes, weakened therapeutic relationships, and hindered conflict management: *[…] I find myself not being able to face that patient who was yelling at me. After everything that happened, you don’t feel comfortable seeing them again.” (Ioannis, 4 years of work experience)*. This discomfort in re-engaging with patients was emblematic of the psychological and relational toll of workplace violence on the participants. It disrupted not only the trust between them and patients, but also the therapeutic alliance, an essential element of quality and safety care.

Career Dissatisfaction and Career Exit Intentions. The cumulative psychological and emotional effects of workplace violence led the participants to consider leaving the profession: *“I don’t want to quit, but yes, there are times when I think about it very strongly.” (Anna, 7 years of work experience).* Prolonged exposure to violence resulted to career dissatisfaction, burnout, and increased thoughts of leaving the profession. Overall, the constant emotional strain and ethical dilemmas created an environment of reduced job satisfaction, contributing to heightened turnover intention.

#### 3.2.3. Coping with Workplace Violence and Strategies for De-Escalation

- Coping with Violence-Related Trauma

Participants reported employing a range of both adaptive and maladaptive coping mechanisms in response to the emotional and psychological toll of workplace violence. Adaptive strategies included leveraging professional experience, cognitive reframing of violent incidents, emotional regulation, and maintaining rigid boundaries between personal and professional life. Social support—particularly from family, friends, and trusted colleagues—served as a critical buffer against psychological distress: *“Given how stressful our work is, it helps to have hobbies that release tension and prevent it from affecting your family.” (Minas, 11 years of work experience).*

Despite these protective strategies, several participants described resorting to maladaptive coping mechanisms, including emotional suppression and substance use, particularly in the absence of institutional support: *“There is so much pressure that it comes out in sleep problems, or in the desire to drink something to relax.” (Christiana, 2 years of work experience).*

- De-escalation Strategies and Self-Protection
  Participants adopted a variety of proactive and reactive strategies to manage violent incidents. Proactively, de-escalation tactics such as calm and measured communication, culturally sensitive negotiation, and the modulation of voice and tone were employed to defuse volatile situations. Where prevention failed, participants turned to practical safety measures including team-based protection, spatial awareness (e.g., remaining near exits), and non-verbal signaling among colleagues: *“You see colleagues trying to keep you out of arguments because you’re new.” (Minas, 11 years of work experience)*
  In severe cases, external intervention was deemed necessary: *“We called the police just so I could be sure I was safe.” (Haris, 6 years of experience).* Importantly, these strategies were often improvised in the absence of formal protocols. The need for standardized, evidence-based training in conflict management and institutional safety planning emerged as a central theme.

#### 3.2.4. Administrative Failure to Manage Workplace Violence and the Hidden Dimensions of Violence Against the Participants

- Institutional Neglect and Minimization of the phenomenon
  A recurring theme in participants’ narratives was institutional failure to acknowledge or appropriately respond to incidents of workplace violence. Several participants reported that administrators downplayed or ignored psychological and verbal abuse, reserving responses solely for physical violence: *“While the hospital may take legal action when there is physical violence, they often turn a blind eye when it’s verbal.” (Anna, 5 years of work experience).* This lack of recognition reinforced a culture of silence and resignation, in which nurses felt compelled to endure violence without recourse.
- Inadequate Safety Infrastructure
  Participants also identified systemic inconsistencies in institutional safety provisions. Security infrastructure such as panic buttons or protected spaces were reportedly available in some departments but absent in high-risk areas, notably emergency departments: *“It’s ironic—some departments have panic buttons, but in the ER, where violence is most likely, we have nothing.” (Ioannis, 4 years of work experience)*

Combined with chronic understaffing and excessive workload, these deficiencies reinforced perceptions of neglect and devaluation of the nursing profession.

#### 3.2.5. Participants’ Expectations and Proposed Interventions

- Institutional and Administrative Reforms
  Participants emphasized the need for comprehensive reforms aimed at enhancing workplace safety. Key recommendations included staff training in de-escalation and trauma-informed care, the implementation of psychological support services, and consistent incident reporting systems. The reinforcement of hospital security and increased staffing were also seen as essential: *“Administrators should provide more security measures and make staff feel safer.” (Ioannis, 4 years of work experience)*
- Organizational Cultural and Leadership Expectations
  There was a strong call for leadership to adopt a unified zero-tolerance stance on violence. Participants argued that even minor incidents should not be dismissed, as such tolerance fosters normalization of aggression: *“We must not tolerate anything—not even a small incident of violence.” (Ioannis, 4 years of work experience)*

Leadership’s role in cultivating a culture of safety and empathy was deemed essential to fostering trust and commitment among nursing personnel.

- Policy-Level Interventions
  Finally, participants recommended structural and legislative changes, including the development of legal frameworks that ensure timely responses to violent incidents and employer accountability. Increased staffing, infrastructure investment, and national standards for violence prevention were seen as crucial to long-term change: *“We need more staff so that the public can be better served.” (Anna, 5 years of work experience)*

## 4. Discussion

Through an exploration of participants’ experiences, this study shed light on the different types of workplace violence and provided critical insights into the enduring and traumatizing effects, also uncovering the institutional and cultural mechanisms that perpetuate workplace violence in the post-pandemic era. This study also showed s that relevant incidents mainly involved patients’ relatives than patients themselves, with verbal abuse emerging as the most prevalent form. To the best to our knowledge, this is the first study to explore the living experience of ED nurses regarding workplace violence in the post-pandemic context placed in the South European and East Mediterranean region.

The COVID-19 pandemic significantly reshaped the landscape of workplace violence in healthcare settings. It aggravated pre-existing vulnerabilities such as staff shortages, increased patient acuity, and public distrust toward the healthcare system [20]. In the post-pandemic years, these adverse dynamics have emphasized rising aggression towards frontline health professionals, particularly in emergency and critical care settings, while healthcare workers continue to struggle with increased stress, burnout, and professional disillusionment [21]. The emerging evidence from the present study confirmed that the intensity and frequency of the phenomenon remain alarming, contributing to its normalization among the participants.

In this context, the present study moved beyond the immediate physical and psychological consequences of violence to examine its long-term implications for professional identity, ethical values, and career trajectories in the participants, while special focus was given on participants’ deteriorating mental health and institutional neglect. Previous studies have extensively documented the negative psychological and professional impact of workplace violence [7,22], often linking it to burnout, job dissatisfaction, and workforce attrition [23,24]. As one of the few post-pandemic studies situating workplace violence within this evolving context [25], the present findings not only confirmed these data but also provided a timely and much-needed analysis that may inform both policy responses and institutional reform. Indeed, international studies showed that rates of violence remained constant or even increased during the COVID-19 pandemic [26,27]; ironically, this came at a time when healthcare workers were being publicly celebrated as ‘heroes’ [28]. Unfortunately, the present study underscored that the extent of phenomenon remains highly disturbing.

Confirming international patterns, the present findings suggest that patients’ relatives more frequently perpetrate violence against healthcare workers; indeed, data show that relatives are responsible for approximately 45–55% of incidents, with patients accounting for the remaining cases [27,29]. Furthermore, verbal abuse was consistently identified as the most common form of violence herein, as it was previously shown for ED and outpatient settings [26,30]. Importantly, the particularly novel contribution of this study regarding the identification of the ‘empathy gap’ between participants and healthcare service users, on one hand, and between participants and administrators—exacerbated by inadequate policies— on the other, expanded the conventional understanding of violence in healthcare. Specifically, the present findings innovatively underscored that administrators, institutional structures and colleagues may play a critical role in either mitigating or amplifying the effects, as well as the triggers of workplace violence on the participants. To our knowledge, there are limited studies that have either identified the aforementioned gap in terms of needs and expectations in individual or in administration level, or at least recognized and discussed it as such in their findings [25,26,31,32,33]. So far, this phenomenon has been emphasized as solely a healthcare service user-related issue, attributing violent incidents to patient-related factors such as mental illness, substance use or high-stress medical environments [8,25,31,32,34]. These elements—with the exception of mental illness—were also confirmed in the present findings.

### 4.1. Instructional Failure and the Impact of Workplace Violence

What emerged as notably damaging for the participants was the lack of institutional support, peer solidarity, and administrative responsiveness to their exposure to violence. This dissonance between the support the participants expected from administrators and colleagues, and the actual response received was deeply traumatic for them. The studies by Gates et al. [35] and Spector et al. [36] previously suggested that institutional failure to address workplace violence exacerbates its impact on healthcare professionals. Following administrators’ unsupportive responses, the participants herein reported feeling abandoned, dismissed, and even blamed by the administrators for the violence they endured, reinforcing their perception that their suffering was invisible or trivialized. This phenomenon of victim blaming by colleagues and administrators further engendered feelings of being unsupported, invalidated, or isolated. Apart from its traumatic impact on the participants, victim blaming was also linked with their inclination to report incidents or seek assistance, as they were apprehensive about being blamed or their claims being disregarded. Uncovering this deficit in support and solidarity within the work settings, the present study also provided concreate evidence that workplace violence is not just a momentary occupational hazard but a sustained and systemic issue reinforced by organizational neglect. Moreover, the failure of administrative measures to address this disconnect and bridge this gap was also seen as a crucial factor in escalating tension within the ED.

These findings are in alignment with the wider literature, which has consistently demonstrated that organizational responses play a crucial role in either mitigating or perpetuating the effects of workplace violence. For instance, Liu et al. [37], in a systematic review and meta-analysis, emphasized that lack of institutional support significantly contributes to the underreporting of incidents, further entrenching a culture of silence and tolerance. Similarly, Vento et al. [26] and Banga et al. [27] reported that healthcare workers often feel disempowered and abandoned by managerial staff when violence occurs, particularly in high-pressure environments such as EDs. Additionally, Sari et al. [29] highlighted that inadequate administrative handling of violence incidents increases staff dissatisfaction and undermines their trust in the system, ultimately affecting their willingness to continue in their job. Moreover, Onal et al. [30] underlined the failure of healthcare institutions in the Eastern Mediterranean region to establish effective reporting and protection mechanisms, noting a direct link with perceived administrative neglect and heightened emotional distress among staff.

This evidence highlighted an urgent ethical and managerial challenge on how institutions may foster a culture that prioritizes healthcare professionals’ well-being and psychological safety, dignity and trust, and subsequently patients’ safety. Formal protocols must not only respond to incidents but actively work to prevent them. Administrators play a pivotal role here; their visibility, empathy and responsiveness can either bridge or widen the empathy gap. Aligned with this consideration, the study by Nouri et al. [38] revealed that the main hindrance to establishing a respectful work environment in nursing was the feeling of being ignored, receiving inadequate feedback and un-addressing problems.

Furthermore, while earlier studies have focused on the physical and emotional toll of violence [22,26,34], the present study uniquely captured how the erosion of empathy within healthcare institutions contributed to professional alienation and moral injury. This negative impact on participants’ empathic attitudes also compromised patient safety. They noted that unresolved exposure to violence and chronic institutional neglect led to emotional detachment, increased irritability and decreased capacity for compassionate care. These dynamics mirror findings from earlier studies suggesting that burnout and emotional fatigue can diminish nurses’ attentiveness and responsiveness [23]. Hence, the consequences of workplace violence extend beyond staff well-being; they pose a direct threat to the quality and safety of patient care.

The dual focus on adaptive and maladaptive coping strategies provided a nuanced understanding of how nurses navigate workplace violence. While previous data support the importance of effective coping mechanisms, generally focusing on resilience-building strategies [39,40], the present study uncovered the darker realities of coping mechanisms, including substance use, emotional detachment, and self-blame alongside the de-escalation attitude “of being the calm in the storm” using techniques of controlled communication and cultural sensitivity, confirming previous data [41]. Although these findings align with recent studies on the hidden psychological costs of workplace violence [22], they mainly extend the conversation by illustrating how coping strategies are shaped by institutional neglect rather than personal shortcomings.

Furthermore, the present findings highlight the complex interplay between systemic and cultural factors in workplace violence. Compared to previous research [25], the present study took a holistic approach, uncovering how gender, physical characteristics, power imbalances and patient-related factors (e.g., intoxication, unrealistic expectations, cultural tensions) intersect to shape the prevalence and severity of experienced violence in the workplace. By doing so, the present study moved beyond simplistic explanations of workplace violence and instead presented it as a deeply embedded structural issue that requires urgent institutional reform, confirming previous data.

### 4.2. Implications for Practice

These findings call for a shift in the discourse from individual resilience to structural accountability. Sustainable solutions must prioritize the development of healthy, supporting coping mechanisms for nurses while holding systems accountable, rather than leaving them to manage trauma in isolation. This requires institutional reform, targeted on two pillars simultaneously—the nurse–service user pillar and the nurse–administrator pillar—by aiming to bridge empathy gaps.

From a policy perspective, previous recommendations emphasize structural and procedural reforms to ensure staff safety and well-being [42]. The American Nurses Association [43] advocates for zero-tolerance policies and peer support systems that not only penalize aggressive patients but also hold administrators accountable for neglecting staff well-being. The enhancement of hospital security infrastructure, including panic buttons and crisis response teams, and adequate staffing access to mental health resources are also proposed [44]. Additionally, mandatory de-escalation and cultural competency training have been considered essential in preventing violence and improving staff preparedness [2,41], while there is a pressing need for stronger legal protections for nurses. Towards this goal, policymakers were considered to support the following: (a) legislation that criminalizes acts of violence against healthcare workers, ensuring swift legal action against offenders, and (b) workplace violence protection laws that require hospitals to meet minimum safety standards and establish formalized intervention protocols [41,42]. Focusing on the under-reporting of violent incidents, an issue clearly reported herein, anonymous reporting mechanisms and clear response protocols to encourage transparency without fear of retaliation are also needed [44]. However, even these will fall short unless paired with cultural change and clear institutional commitment. Reporting should not be seen as a risk but as a protected right and professional responsibility, reinforced by leadership.

Despite the importance of these interventions, the empathy gap between nurses and patients/service users must be addressed with equal urgency. Unrealistic expectations, miscommunication, and cultural disconnects were recurring themes in our data. Interventions designed to reduce violence must include education for patients and families about healthcare system constraints, staff limitations, and acceptable behavior. For instance, monitors and leaflets in waiting rooms explaining the routine of the ED, related procedures and expected waiting times may result in enhanced understanding in service users about nurses’ tasks and delays in receiving care. Nurses, in turn, must be supported with communication tools and structured spaces for emotional reflection. Interventions that cultivate mutual respect—through workshops, co-designed policy development, or empathy-building campaigns—are likely to yield longer-lasting outcomes. Empathy is not merely an interpersonal trait; it is a structural requirement. Its absence was identified by participants not only as a cause of violence but as a reason for its escalation and persistence.

Overall, the present findings support the articulation of interventions developed on the basis of a participatory approach, with focus on how to bridge the empathy gap between stakeholders. Thus, relevant projects need to include all three groups of them, i.e., nurses, patients and the families, and administrators, aiming to enhance each groups’ empathic understanding, and cultivate a culture of civility. By fostering empathy and open communication, such interventions may not only improve the psychological safety of nurses but also contribute to greater patient satisfaction and reduce aggression in clinical encounters, all resulting in better clinical outcomes.

### 4.3. Strengths and Limitations

The key strength of the present study was its post-pandemic perspective, as it acknowledged that workplace violence cannot be examined in isolation from broader societal changes. By situating workplace violence within this evolving context, this study provided a timely and urgently needed analysis that may inform both policy responses and institutional reforms. Additionally, the revelation of the “empathy gap” as a core theme strengthens the impact of the study. This finding is expected to challenge the conventional understanding that violence in healthcare is solely a patient-related issue [8]; instead, the present findings showed that colleagues, administrators and institutional structures play a critical role in the dynamic of the phenomenon.

Regarding the limitations of the present study, one may highlight the sample size of six participants as a limitation regarding the extent to which these findings may be transferred to the broader population. Similarly, the unique characteristics of the healthcare system of the RC may restrict the transferability of the findings to other contexts. However, the data and theoretical saturation that were achieved herein support the credibility, dependability and confirmability of the study [15]. Indeed, theoretical saturation was achieved, as no new codes or themes emerged during the final interviews. This is consistent with guidance from Guest et al. [18], who found that saturation can occur within the first six interviews when the study focus is clear and participants share common experiences. In our case, all participants were ED nurses operating under similar organizational structures and reporting analogous experiences of workplace violence, supporting thematic redundancy. Nonetheless, the limited sample size is acknowledged as a methodological constraint, and future research with a broader and more diverse sample is recommended to enhance the comprehensiveness and transferability of the findings [45]. The absence of contradictory or disconfirming data may also reflect the relative homogeneity of the participant group, as all of them shared similar roles, work environments, and exposure to workplace violence. While this consistency reinforces the coherence and dependability of the findings, it may limit the diversity of perspectives represented. The influence of researchers’ positionality and possible selection bias in participants’ recruitment also need to be considered.

On the other hand, the exclusive reliance of data collection on personal interviews may pose restrictions on the depth of understanding which could have been achieved through data triangulation with other methods, such as observation or journal keeping. Yet, the participants provided insightful narratives following comprehensive reflection of their experiences, as was shown by the extent of the paradigms provided and the dimensions of the phenomenon described. Additionally, the potential recall bias among participants when recounting past experiences could have influenced the accuracy of the data collected. To address this limitation, during the repeated interviews, the participants had the chance to enrich and confirm their narratives. Furthermore, since English was not the researchers’ mother language, the accuracy of transferring participants’ data from their native language to the present form may have biased their interpretation. Aiming to eliminate this limitation, the translation of quotes was confirmed by a software for professional translation and was further validated by a bilingual research associate. Nevertheless, the strength of the present study may be attributed to the insightful experiences and subsequent powerful narratives of the participants described herein for the first time within this particular cultural context. This adds important and novel information to the current literature on the subject. Most importantly, since the participants’ findings are based on both data and theoretical saturation, they may be deemed as representative of the target population and its work-related and cultural context from which they were collected, i.e., Greek-speaking emergency nurses employed in public and private settings in the RC. Indeed, the present analysis was based on a multi-layered approach, which integrated qualitative data from a diverse sample of emergency nurses. By capturing the living experiences of nurses with varying levels of expertise (2–12 years) and across different departments coming from both the public and private sector, the current study provides a dynamic and intersectional understanding of workplace violence. The qualitative depth of the narratives allowed for a more humanized exploration of violence, highlighting not only its external manifestations but also its internalized consequences, such as self-doubt, moral distress and career dissatisfaction.

## 5. Conclusions

This study contributes a critical post-pandemic perspective on workplace violence, highlighting not only its psychological and professional impact but also the institutional failures that sustain it. By identifying the empathy gap as a core theme, this research challenges the prevailing discourse that views violence as an inevitable occupational risk, instead framing it as a preventable systemic and relational failure. To bridge these divides, future intervention studies should examine strategies that cultivate empathy across all levels, including trauma-informed care training, patient–staff communication frameworks, and organizational practices that value emotional intelligence. Administrators should implement policies that go beyond punitive responses, emphasizing transparent reporting, proactive staff support, and patient education initiatives. By addressing both institutional and interpersonal dimensions of empathy, healthcare systems can better safeguard the safety, dignity, and sustainability of emergency nursing.

## Figures and Tables

**Table 1:** Presentation of the criteria applied to ensure the rigor and trustworthiness of the study [18].

**Criterion**	**Description**	**How the Criterion Was Supported in the Study**
Reasonableness	The results constitute a reasonable interpretation of the phenomenon	The findings were confirmed (a) in the phase of repeated interviews, and (b) during the presentation of the findings to the participants; Participants’ expressions such as “Yes, it seems logical to me,” and “Yes, it makes sense to me” were deemed as confirmatory statements. The integrity of the data analysis process was assured by continuous meetings of the three researchers in order to determine the degree of consensus among them regarding the findings. In the event of any differences in opinion, the researchers engaged in extensive dialogue to reach a consensus. Ultimately, all three researchers agreed regarding the study’s findings.
Resonance	The analysis of the phenomenon reverberates with participants	The analysis was corroborated through participants’ feedback during iterative interviews and findings’ presentations. This was evidenced by statements such as “Yes, that accurately reflects my perspective.” Moreover, through repetitive meetings with each participant, the researcher allowed sufficient time to build a relationship which facilitated the sharing of experiences.
Representativeness	In depth interpretation of the phenomenon	Aiming to ensure data saturation, the following measures were implemented: (a) participants came from both public and private hospitals, they had adequate work experience as an ER nurse, both genders and diverse family status (single/divorced/married) were represented equally, (b) participants were prompted to illustrate their experiences with concrete examples, thereby fostering a comprehensive understanding of their meaning, (c) interviews were concluded only when participants indicated they had exhausted their responses, using a closing query such as, “Is there anything further you’d like to include?”, (d) the time spent on data collection and analysis was sufficient, considering the sample size, the complexity of the study design, and the scope of the phenomenon, and (e) all participants engaged in a follow-up interview, allowing for reflection and potential expansion or revision of their initial accounts- by conducting supplementary interviews and spending time for deepening the understanding of the data during analysis and interpretation the degree of data and theoretical saturation were enhanced. Additional measures were followed, aiming to reach theoretical saturation. Specifically, data collection and analysis took place simultaneously allowing for the identification of recurrent themes, while data collection was ended when additional narratives and further analysis did not contribute to the emerging themes.
Relevance	The relevance of the study to the objectives of the qualitative paradigm	The study’s conclusion draws parallels between the experiences of violence among participants and their self-perceptions, as revealed through their narratives.
Recognizability	Readers recognize aspects of their experience in the interpretation	The applicability of the findings regarding the consequences of violence against nurses was confirmed through the presentation of these findings to nurses from different healthcare settings and to healthcare administrators, citing expressions such as: ‘What you are describing to me, I have witnessed firsthand in my department’.

**Table 2:** Presentation of the categories, themes and sub-themes of the present analysis.

**Core Theme**	**Categories**	**Themes**	**Sub-Themes**
***The divergence in needs and the pronounced empathy gap between (a) participants and patients/family members-caregivers, and (b) the participants’ needs and administrators’ attitudes and related policies; administrative measures failure to bridge the empathy gap, contributing to tension escalation in the ED.***	**The surreal experience of WV: forms, interpretations and characteristics**	Participants’ awareness of WV	
		The surreal nature of WV	
		Patterns and forms of WV	
		Violation of boundaries: a profound lack of empathy and respect	
		Normalization of WV	
		Persecution, institutional neglect and lack of protection	
		Contributing factors to WV	- Individual-level risk factors
			- Patient-related triggers
	**Experiencing WV: the traumatic and multifaceted impact on the participants**	Personal-level impact	- Mental effects
			- Physical effects
			- Psychological effects
			- Self-perception, personal values and morality
		Social-level impact	
		Professional-level impact	- Professional competence.
			- Professional identity
			- Ethical and emotional strain
			- Disruption of healthcare processes and patient care
			- Career dissatisfaction and career exit intentions
	**Coping with WV and strategies for de-escalation**	Coping with violence-related trauma	
		De-escalation strategies and self-protection	
	**Administrative failure to manage WV and the hidden dimensions of violence against the participants**	Institutional neglect and minimization of the phenomenon	
		Inadequate safety infrastructure	
	**Participants’ expectations and proposed interventions**	Institutional and administrative reforms	
		Organizational cultural and leadership expectations	
		Policy-level interventions	
**WV**: Workplace Violence			

## Data Availability

The data supporting the findings of this study are available from the corresponding author upon reasonable request.

## Supplementary Materials

The following supporting information can be downloaded at: https://www.mdpi.com/article/10.3390/healthcare13101118/s1, Table S1: Assessment of the rigor of the present study according to the CASP tool; Table S2: The COREQ tool assessment as it was applied in the present study.

## Abbreviations

The following abbreviations are used in this manuscript:

ED	Emergency Department
WV	Workplace Violence
ER	Emergency Room
ANA	American Nurses Association
AHA	American Hospital Association
RC	Republic of Cyprus
