# Supplementary material for: “Divergent Needs and the Empathy Gap”: Exploring the Experience of Workplace Violence Against Nurses Employed in the Emergency Department
Source: Healthcare (Basel). 2025 May 11;13(10):1118. doi: 10.3390/healthcare13101118 (PMC12111539; doi:10.3390/healthcare13101118)
Supplement: Supplementary file 1 [file healthcare-13-01118-s001.zip › healthcare-3609566-supplementary.pdf]

CASP Checklist:
For Qualitative Research

Reviewer Name:	
Paper Title:	"Divergent Needs and the Empathy Gap": Exploring the Experience of Workplace Violence Against Nurses Employed in the Emergency Department
Author:	Christina Koutsofta, Dr. Maria Dimitriadou, Dr. Maria Karanikola
Web Link:	
Appraisal Date:	

During critical appraisal, never make assumptions about what the researchers have done. If it is not possible to tell, use the "Can't tell" response box. If you can't tell, at best it means the researchers have not been explicit or transparent, but at worst it could mean the researchers have not undertaken a particular task or process. Once you've finished the critical appraisal, if there are a large number of "Can't tell" responses, consider whether the findings of the study are trustworthy and interpret the results with caution.

Table S1 Assessment of the rigor of the present study according to the CASP tool

Section A Are the results valid?	
1. Was there a clear statement of the aims of the research?<br><br>The study clearly stated its aim to explore the lived experiences of exposure to violence from healthcare service users and the consequences in there three levels of life, personal, social and professional level.	☒Yes ☐ No ☐ Can't Tell
CONSIDER:<br>• *what was the goal of the research?*<br>• *why was it thought important?*<br>• *its relevance*	
2. Is a qualitative methodology appropriate?<br><br>The study sought to understand the subjective experiences of the nurses in ER, making a qualitative approach appropriate.	☒Yes ☐ No ☐ Can't Tell
CONSIDER:<br>• *If the research seeks to interpret or illuminate the actions and/or subjective experiences of research participants*<br>• *Is qualitative research the right methodology for addressing the research goal?*	
3. Was the research design appropriate to address the aims of the research?<br><br>The study employed semi-structured interviews which allowed for in-depth exploration of individual experiences.	☒Yes ☐ No ☐ Can't Tell
CONSIDER:<br>• *if the researcher has justified the research design (e.g., have they discussed how they decided which method to use)*	
4. Was the recruitment strategy appropriate to the aims of the research?<br><br>Convenience sampling was conducted so that only the target population of the research question participated. Nurses from other departments were not selected for participation due to the focus of the study on Emergency department for research purposes.	☒Yes ☐ No ☐ Can't Tell

CONSIDER:<br>• *If the researcher has explained how the participants were selected*<br>• *If they explained why the participants they selected were the most appropriate to provide access to the type of knowledge sought by the study*<br>• *If there are any discussions around recruitment (e.g. why some people chose not to take part)*	
5. Was the data collected in a way that addressed the research issue?<br><br>The interviews were conducted by trained researcher. The interview guide was developed by the researchers after an extensive literature review, aiming to address the research question and the objectives of the present study. The study employed semi-structured interviews which allowed for in-depth exploration of individual experiences.	☒Yes ☐ No ☐ Can't Tell
CONSIDER:<br><br>• *If the setting for the data collection was justified*<br>• *If it is clear how data were collected (e.g. focus group, semi-structured interview etc.)*<br>• *If the researcher has justified the methods chosen*<br>• *If the researcher has made the methods explicit (e.g. for interview method, is there an indication of how interviews are conducted, or did they use a topic guide)*<br>• *If methods were modified during the study. If so, has the researcher explained how and why*<br>• *If the form of data is clear (e.g. tape recordings, video material, notes etc.)*<br>• *If the researcher has discussed saturation of data*	
6. Has the relationship between researcher and participants been adequately considered?<br><br>The researchers acknowledged their potential biases and took steps to minimize their influence on the participants. The main researcher, after daily reflection, had a complete conscience in order to ensure the impartiality of the results after each interview.	☒Yes ☐ No ☐ Can't Tell
CONSIDER:<br>• *If the researcher critically examined their own role, potential bias and influence during (a) formulation of the research questions (b) data collection, including sample recruitment and choice of location*	

• *How the researcher responded to events during the study and whether they considered the implications of any changes in the research design*	
Section B: What are the results?	
7. Have ethical issues been taken into consideration?<br><br>The study obtained ethical approval and ensured informed consent from all participants, protecting their anonymity and confidentiality. Along with the consent form, an information leaflet was given for full understanding by the participants. Participants were also invited to provide information about their age, gender, and years of experience in the ER. Interviews were conducted individually in private settings outside hospital premises, during participants' free time, to ensure confidentiality, minimize potential stigma, and prevent interference with their professional environment.<br>The protocol for the research was registered and approved by the ethical committee of Cyprus National Bioethics Committee (ΕΕΒΚ ΕΠ 2023.01.219). To ensure confidentiality, no participant names were used in any audio recording or transcripts.	☒Yes ☐ No ☐ Can't Tell
CONSIDER:<br>• *If there are sufficient details of how the research was explained to participants for the reader to assess whether ethical standards were maintained*<br>• *If the researcher has discussed issues raised by the study (e.g. issues around informed consent or confidentiality or how they have handled the effects of the study on the participants during and after the study)*<br>• *If approval has been sought from the ethics committee*	
8. Was the data analysis sufficiently rigorous?<br><br>The study employed content analysis, a well-established method for analysing qualitative data. To ensure transparency and rigor, the study employed the triangulation method. The content analysis team independently analysed each interview. Then, the team reviewed and validated the codes and themes generated by each researcher. Then the categories were developed. With this technique, there was an exchange of opinions, and the transparency of the results was ensured, due to the involvement of many perceptions of the content of the interviews, and it would be difficult to lose any data. The involvement of multiple perspectives strengthened the rigor and trustworthiness of the results.	☒Yes ☐ No ☐ Can't Tell

Content analysis process began immediately after one or two interviews, with the researcher transcribing audio recordings into written format to prepare for coding. The content analysis team comprised the principal researcher and three academic collaborators, who jointly reviewed and coded the data. Before starting content analysis, the researcher recorded the taped interview in paper form so that it was ready for coding. Through teamwork, code groups were established reflecting the participants' common interpretations, descriptions, and perceptions of the data. This collaborative process facilitated the emergence of key categories and themes.	
CONSIDER:<br>• *If there is an in-depth description of the analysis process*<br>• *If thematic analysis is used. If so, is it clear how the categories/themes were derived from the data*<br>• *Whether the researcher explains how the data presented were selected from the original sample to demonstrate the analysis process*<br>• *If sufficient data are presented to support the findings*<br>• *To what extent contradictory data are taken into account*<br>• *Whether the researcher critically examined their own role, potential bias and influence during analysis and selection of data for presentation*	
9. Is there a clear statement of findings?<br><br>The study presented its findings in a clear and organized manner, using quotes from participants to illustrate the themes. Six categories emerged, with themes and sub-themes for each category. To ensure transparency and rigor, the study employed the triangulation method. The content analysis team independently analysed each interview.	☒ Yes ☐ No ☐ Can't Tell
CONSIDER:<br>• *If the findings are explicit*<br>• *If there is adequate discussion of the evidence both for and against the researcher's arguments*<br>• *If the researcher has discussed the credibility of their findings (e.g. triangulation, respondent validation, more than one analyst)*<br>• *If the findings are discussed in relation to the original research question*	
Section C: Will the results help locally?	
10. How valuable is the research?	☒ Yes ☐ No ☐ Can't Tell

The study provides valuable insights into the experiences of Violence survivors.<br>Recognizing and addressing violent behaviours towards nurses, as well as providing information about the effects of violence, will benefit the well-being and health of nurses, the quality and safety of care, and the sustainability of organizations. This study explores the experience of violent behaviour and its impacts, placing Cyprus within a broader cultural context of the East Mediterranean region.<br>Hospitals can implement programs offering psychological counselling, peer support groups, and debriefing sessions after violent incidents. The findings also emphasize the importance of recognizing the impact of violence on nurses' personal, social, and professional lives, leading to more holistic support.	
CONSIDER:<br>• *If the researcher discusses the contribution the study makes to existing knowledge or understanding (e.g., do they consider the findings in relation to current practice or policy, or relevant research-based literature*<br>• *If they identify new areas where research is necessary*<br>• *If the researchers have discussed whether or how the findings can be transferred to other populations or considered other ways the research may be used*	

Table S2: The COREQ tool assessment as it was applied in the present study

**APPRAISAL SUMMARY**: *List key points from your critical appraisal that need to be considered when assessing the validity of the results and their usefulness in decision-making.*		
**Positive/Methodologically sound**	**Negative/Relatively poor methodology**	**Unknowns**
**Clear aim**<br>**Appropriate methodology**<br>**Rigorous data collection**<br>**Thorough data analysis**<br>**Ethical considerations**<br>**Rich data**		
